# Increased Mortality among Survivors of Myocardial Infarction with Kidney Dysfunction: the Contribution of Gaps in the use of Guideline-Based Therapies

**DOI:** 10.1186/1471-2261-9-29

**Published:** 2009-07-08

**Authors:** Pamela N Peterson, Amrut V Ambardekar, Philip G Jones, Harlan M Krumholz, Erik Schelbert, John A Spertus, John S Rumsfeld, Frederick A Masoudi

**Affiliations:** 1 Denver Health Medical Center, Denver, Colorado, USA; 2 University of Colorado Denver, Aurora, Colorado, USA; 3 Mid America Heart Institute of Saint Luke's Hospital, Kansas City, Missouri, USA; 4 Yale, New Haven, Connecticut, USA; 5 National Institutes of Health, Bethesda, Maryland, USA; 6 University of Missouri – Kansas City, Kansas City, Missouri, USA; 7 Denver VA Medical Center, Denver, Colorado, USA

## Abstract

**Background:**

We assessed the degree to which differences in guideline-based medical therapy for acute myocardial infarction (AMI) contribute to the higher mortality associated with kidney disease.

**Methods:**

In the PREMIER registry, we evaluated patients from 19 US centers surviving AMI. Cox regression evaluated the association between estimated glomerular filtration rate (GFR) and time to death over two years, adjusting for demographic and clinical variables. The contribution of variation in guideline-based medical therapy to differences in mortality was then assessed by evaluating the incremental change in the hazard ratios after further adjustment for therapy.

**Results:**

Of 2426 patients, 26% had GFR ≥ 90, 44% had GFR = 60- < 90, 22% had GFR = 30- < 60, and 8% had GFR < 30 ml/min/1.73 m^2^. Greater degrees of renal dysfunction were associated with greater 2-year mortality and lower rates of guideline-based therapy among eligible patients. For patients with severely decreased GFR, adjustment for differences in guideline-based therapy did not significantly attenuate the relationship with mortality (HR 3.82, 95% CI 2.39–6.11 partially adjusted; HR = 3.90, 95% CI 2.42–6.28 after adjustment for treatment differences).

**Conclusion:**

Higher mortality associated with reduced GFR after AMI is not accounted for by differences in treatment factors, underscoring the need for novel therapies specifically targeting the pathophysiological abnormalities associated with kidney dysfunction to improve survival.

## Background

Despite advances in treatment, patients with kidney disease experience worse outcomes than patients with normal kidney function following acute myocardial infarction (AMI) .[1-3] Furthermore, among community based populations with AMI, as many as half of patients have moderate to severe kidney disease.[1,4-6] Given the high prevalence of kidney disease in patients with AMI, it is important to understand the mechanisms underlying the higher rates of adverse outcomes in this population.

The excess risk associated with kidney dysfunction in patients with AMI may result from biologic factors as well as differences in treatment with guideline-recommended therapies. Several pathophysiological mechanisms have been proposed to explain the progressive increase in risk with worsening kidney function, including oxidative stress, inflammation, elevated fibrinogen levels and derangements in calcium-phosphate homeostasis.[7,8] Beyond biologic mechanisms, the lower use of therapies in patients with kidney dysfunction may also contribute to their higher morbidity and mortality. Although studies have consistently demonstrated lower use of guideline-based therapies among patients with kidney dysfunction and AMI,[1,2,6,9-13] the contribution of this variation in care to differences in outcomes has been assumed but not directly assessed.

The objective of this study was to evaluate the contribution of differential medical treatment to the association between kidney function and mortality in a community-based contemporary cohort of post-AMI patients. We hypothesized that differences in guideline-based medical therapy would, at least in part, account for the higher mortality associated with kidney dysfunction. Understanding the extent to which differences in treatment explain differences in outcomes would help define the importance of efforts to increase the use of guideline-based therapies compared with those to develop treatments targeting the pathophysiological abnormalities conferred by kidney dysfunction in AMI patients.

## Methods

### Study Population

Patients were enrolled in the Prospective Registry Evaluating Myocardial Infarction: Event and Recovery (PREMIER) study, a prospective cohort study of the care and outcomes of patients after AMI. PREMIER enrolled patients from 19 US Centers between January 2003 and June 2004.[14] All patients with a positive troponin test or elevated CPK-MB fractions were screened for possible inclusion.

Eligible patients were ≥ 18 years of age, had either an elevated troponin or CPK-MB, had other supporting evidence suggestive of an AMI (e.g. prolonged ischemic symptoms, electrocardiographic ST changes) and presented at the enrolling institution or were transferred within the first 24 hours of symptom onset (to ensure that primary clinical decision making occurred at the enrolling site). Patients provided informed consent, approved by the human subjects review boards at each institution, to participate.

**Figure 1:**
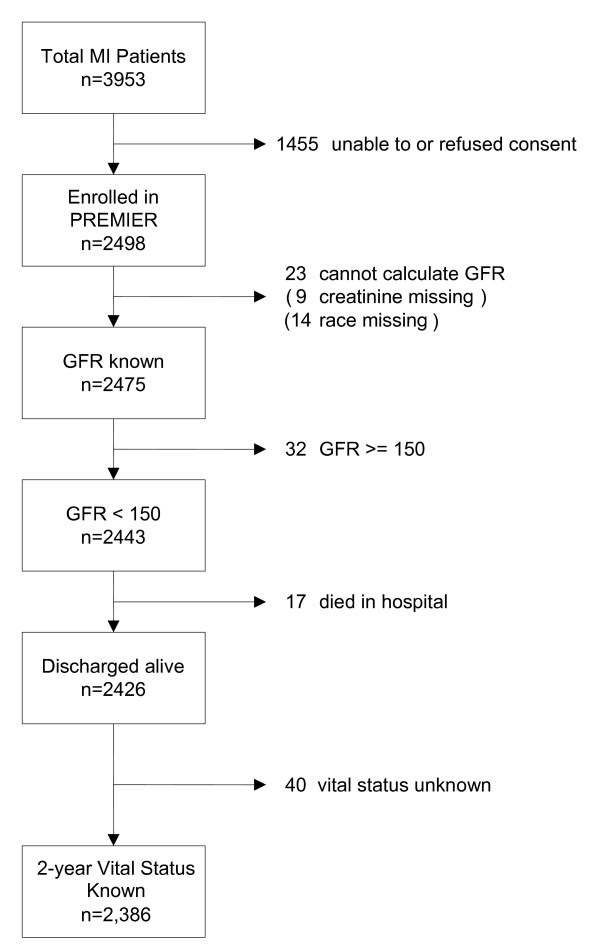
**Patient accounting**.

Of the 2,498 patients enrolled in PREMIER, 2,426 survived to discharge and had complete data to calculate an estimated glomerular filtration rate (GFR). (Figure 1)

### Variables

The primary predictor variable was kidney function as quantified by the GFR as calculated using the four variable Modification of Diet in Renal Disease (MDRD) forumla.[15] The creatinine used for this calculation was the measurement closest to the time of hospital discharge. The GFR was categorized according to recommendations of the National Kidney Foundation-Kidney Disease Outcomes Quality Initiative as follows: normal = GFR ≥ 90 mL/min/1.73 m^2^; mildly impaired = GFR 60- < 90 mL/min/1.73 m^2^; moderate dysfunction = GFR 30- < 60 mL/min/1.73 m^2^; and severe dysfunction = GFR < 30 mL/min/1.73 m^2^.[16]

A wide range of demographic, cardiac, non-cardiac, and treatment factors were used for risk adjustment. Records were abstracted by trained reviewers for clinical comorbidities, admission medications, presenting ECG findings, diagnostic study results, final diagnoses, medical therapies and procedures. Specific medical therapies of interest were treatment during the first 24 hours with aspirin and beta-blockers and discharge prescription of aspirin, beta-blockers, statins, and angiotensin converting enzyme inhibitors (ACE inhibitors) or angiotensin receptor blockers (ARBs). Patients were considered eligible for aspirin, beta blockers and statins if a contraindication or intolerance to therapy was not documented in the medical record. Patients considered eligible for ACE inhibitors or ARBs, were those with at least moderate left ventricular systolic dysfunction and no contraindication or intolerance to therapy documented in the medical record. Contraindications and intolerance to treatments were collected in a prospective fashion. Contraindications were not explicitly defined, but rather determined to be present and documented in the medical record by the treating clinician.

The primary outcome was time to death. Vital status was confirmed by means of telephone contact and a query of the National Death Index. Vital status was available for 98% of patients (n = 2,386) at two years. The proportion missing vital status at two years did not differ by GFR.

### Statistical Analysis

Baseline demographic and clinical variables were compared among groups according to categories of GFR using chi square tests for trends for categorical variables and ANOVA for continuous variables. The unadjusted association between kidney function and treatment rates was evaluated by comparing proportions of eligible candidates receiving therapy in categories of GFR using chi square tests. Because of the small numbers of patients with ESRD, patients receiving hemodialysis were included in the category of severe kidney dysfunction in the primary analysis. In secondary analyses of treatment rates, dialysis patients were considered separately and treatment rates were compared across all categories of kidney function.

To evaluate the unadjusted association between categories of GFR and mortality at two years, Kaplan-Meier survival curves were constructed and were compared using the log rank test. Multivariable Cox proportional-hazard regression, stratified by site, was then used to evaluate the risk-adjusted association between GFR categories and mortality. A multivariable model was first constructed including demographic and clinical variables (age, gender, race, body mass index (BMI), smoking, cocaine use, alcohol use, diabetes, hypercholesterolemia, hypertension, peripheral arterial disease, stroke, chronic lung disease, arthritis, cancer, prior MI, prior percutaneous coronary intervention (PCI), prior coronary artery bypass graft (CABG), heart failure, left ventricular systolic function, type of MI, maximum troponin, receipt of adjunctive antiplatelet therapy, receipt of anti-thrombin therapy, primary reperfusion for ST elevation MI (STEMI), revascularization with PCI, revascularization with CABG). Because not all patients are eligible for medical therapies (e.g. some have a documented contraindication or intolerance to treatment), eligibility may differ across categories of GFR, and eligibility for therapy may be associated with differences in mortality, we next adjusted for eligibility for aspirin, beta blockers, statins, and ACE inhibitor or ARB. Finally, the use of guideline-based medical therapy with aspirin (at admission and discharge), beta blockers (at admission and discharge), statins (at discharge) and ACE inhibitors or ARBs (at discharge) were added to the model. The mediation proportion or the incremental contribution of receipt of medications to the observed association between categories of GFR and mortality was evaluated by comparing the hazard ratios for the relationship between kidney function and mortality before and after adjustment for treatment with medications.

To further assess whether or not the differences in mortality in categories of GFR were due to differences in treatment, separate models were run restricted to those eligible for each therapy. Within the cohort restricted to those eligible, the hazard ratios for the relationship between categories of GFR and mortality before and after adjustment for treatment with medication were then compared. All analyses were conducted using SAS software, Version 9.1 (SAS Institute Inc., Cary, NC).

The authors had full access to the data and take responsibility for its integrity.

## Results

Of the 2426 patients, 26% (626) had GFR ≥ 90 ml/min/1.73 m2, 44% (1073) had GFR 60- < 90 ml/min/1.73 m2, 22% (530) had GFR 30- < 60 ml/min/1.73 m2, and 8% (197) had GFR < 30 ml/min/1.73 m2 or were on hemodialysis (n = 76). Compared with patients with a normal GFR, patients with lower GFR were older, had lower BMI, had a higher prevalence of comorbidities, and were more likely to have had a prior MI, prior CABG and prior PCI (Table 1). Patients with reduced GFR were more likely to present with a non-ST elevation MI (NSTEMI) and to have had higher TIMI risk scores as compared with patients with normal GFR. Of those who presented with STEMI, patients with kidney dysfunction were less likely to receive reperfusion therapy with primary PCI, fibrinolysis or immediate CABG. Additionally, those with reduced GFR less frequently underwent coronary angiography or coronary procedures at any time during their hospitalization.

**Table 1:** Baseline characteristics of the population by categories of estimated glomerular filtration rate.

**Variable**	**GFR ≥ 90 (n = 626)**	**GFR 60- < 90 (n = 1073)**	**GFR 30- < 60 (n = 530)**	**GFR < 30 (n = 197)**	**p (trend)**
Age, mean years (SD)	54 (11)	61 (13)	68 (11)	63 (14)	< 0.001
Male (%)	75	71	54	56	< 0.001
Race					
White (%)	68	82	78	42	< 0.001
Black (%)	26	14	17	53	
Other (%)	6	4	5	5	
***Non-Cardiac History***					
BMI, mean kg/m^2 ^(SD)	27.6 (6.1)	29.1 (6.3)	29.3 (6.2)	29.7 (6.7)	0.003
Diabetes (%)	24	22	38	57	< 0.001
Hypercholesterolemia (%)	43	52	52	48	0.002
Hypertension (%)	53	60	77	87	< 0.001
PAD (%)	4	6	11	22	< 0.001
Prior stroke (%)	4	5	10	12	< 0.001
Chronic lung disease (%)	11	12	16	21	< 0.001
***Cardiac History***					
MI (%)	18	20	27	28	< 0.001
CABG (%)	9	10	18	25	< 0.001
PCI (%)	15	17	22	21	0.006
Heart Failure (%)	6	7	19	39	< 0.001
LV Function: normal/mild (%)	76	78	68	63	< 0.001
Type of MI (%)					
STEMI	49	49	34	11	< 0.001
NSTEMI	51	50	65	88	
TIMI STEMI Score (SD)	2.6 (1.8)	3.1 (2.1)	4.7 (2.3)	4.5 (2.5)	< 0.001
TIMI NSTEMI Score (SD)	2.8 (1.3)	3.2 (1.3)	3.6 (1.3)	3.3 (1.4)	< 0.001
Acute Reperfusion (%)	67	71	49	15	< 0.001
Revascularization (%)	80	78	62	32	< 0.001
***Coronary Angiography***					
All (%)	93	93	82	50	< 0.001
STEMI (%)	96	96	90	60	< 0.001
NESTEMI (%)	89	90	76	47	< 0.001
***Laboratory***					
Hemoglobin (g/dL): mean (SD)	14.2 (2.6)	13.9 (1.9)	13.0 (2.1)	11.3 (2.3)	< 0.001
Creatinine (mg/dL): mean (SD)	0.9 (0.4)	1.1 (0.4)	1.4 (0.5)	5.1 (3.8)	< 0.001

**Table 1 abbreviations**: CABG = coronary artery bypass graft surgery; GFR = glomerular filtration rate (ml/min/1.73 m2); MI = myocardial infarction; LV = left ventricular; NSTEMI = non-ST-segment elevation myocardial infarction; PAD = peripheral arterial disease; PCI = percutaneous coronary intervention; STEMI = ST segment elevation myocardial infarction; TIMI = Thrombolysis in Myocardial Infarction

The proportion of patients eligible for each therapy by GFR is shown in Table 2. Among those who were eligible (no documented allergy or contraindication), patients with reduced GFR were significantly less likely to receive guideline-based therapies (Table 3). Differences in the rate of therapy were most marked for aspirin within 24 hours of admission, aspirin at discharge, statins at discharge, and ACE inhibitor or ARB at discharge. The rate of acute and discharge beta-blocker therapy did not differ across categories of GFR. Of note, in secondary analyses, among those who were on dialysis, 89% received aspirin within 24 hours (p-value for trend across categories of renal function = 0.003), 80% received aspirin at discharge (p-value for trend < 0.001), 67% received a statin at discharge (p-value for trend < 0.001), and 64% received an ACE inhibitor or ARB (p-value for trend < 0.001).

**Table 2:** Proportions of patients eligible for guideline-based therapies by categories of estimated glomerular filtration rate.

**Treatment**	**GFR ≥ 90 (n = 626)**	**GFR 60- < 90 (n = 1073)**	**GFR 30- < 60 (n = 530)**	**GFR < 30 (n = 197)**	**p (trend)**
Acute aspirin	99.0%	98.5%	97.9%	98.0%	0.114
Acute beta blocker	93.6%	95.6%	93.4%	92.9%	0.531
Discharge aspirin	98.4%	97.6%	96.2%	94.4%	0.001
Discharge beta blocker	94.6%	96.0%	96.2%	95.9%	0.220
Discharge ACE/ARB	22.5%	20.1%	29.1%	27.4%	0.006
Discharge Statin	97.4%	97.3%	95.7%	95.9%	0.073

GFR = glomerular filtration rate (ml/min/1.73 m2)

**Table 3:** Proportions of eligible patients receiving guideline-based therapies by categories of estimated glomerular filtration rate.

**Treatment**	**GFR ≥ 90 (n = 626)**	**GFR 60- < 90 (n = 1073)**	**GFR 30- < 60 (n = 530)**	**GFR < 30 (n = 197)**	**p (trend)**
Acute aspirin	98%	96%	97%	93%	0.019
Acute beta blocker	94%	92%	90%	89%	0.166
Discharge aspirin	94%	95%	92%	86%	< 0.001
Discharge beta blocker	91%	93%	91%	89%	0.389
Discharge ACE/ARB	84%	92%	81%	56%	< 0.001
Discharge statin	83%	84%	76%	69%	< 0.001

GFR = glomerular filtration rate (ml/min/1.73 m2)

**Figure 2:**
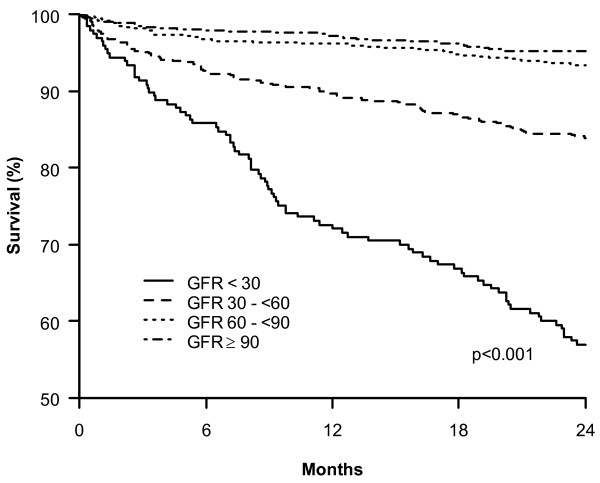
**Kaplan-Meier survival curve by estimated glomerular filtration rate (GFR, ml/min/1.73 m2)**.

Overall, patients with reduced GFR had higher mortality rates at two years than patients with normal GFR (Figure 2). A progressive increase in mortality was observed with decreasing GFR (3% for those with GFR > 90 ml/min/1.73 m2, 4% for GFR 60- < 90 ml/min/m2; 10% for GFR 30- < 60 ml/min/m2 and 28% for GFR < 30 ml/min/m2, p < 0.001).

After adjustment for demographics, comorbidities, adjunctive thienopyridine therapy, reperfusion and revascularization and eligibility for medical therapies, compared to those with GFR ≥ 90, there was a trend toward increased 2-year mortality for patients with GFR of 60- < 90 ml/min/m2 (HR = 1.24; 95% CI 0.78–1.97) and GFR of 30- < 60 ml/min/m2 (HR = 1.56; 95% CI 0.96–2.54) and significantly higher mortality for those with GFR < 30 ml/min/m2 (HR 3.45; 95% CI 2.11–5.64) (Table 4). Further adjusting for differences in treatment patterns for aspirin, beta blockers, statins and ACE inhibitors or ARBs did not attenuate the association with mortality for patients with GFR of 60- < 90 ml/min/m2 (HR 1.24; 95% CI 0.78–1.97), GFR 30- < 60 ml/min/m2 (HR 1.56; 95% CI 0.96–2.56), or GFR < 30 ml/min/m2 (3.56; 95% CI 2.16–5.87) (Table 4). The results were similar for each individual therapy when models were restricted to only those eligible for that therapy.

**Table 4:** Hazard ratios (95% confidence intervals) for the relationship between estimated glomerular filtration rate and mortality.

**Adjustment:**	**GFR ≥ 90**	**GFR 60–89**	**GFR 30–59**	**GFR < 30**
Unadjusted	ref	1.44 (0.93–2.25)	3.35 (2.16–5.20)	7.52 (4.85–11.66)
Demographic and clinical variables, + reperfusion/revascularization, adjunctive antiplatelet therapy	ref	1.23 (0.77–1.95)	1.51 (0.93–2.46)	3.35 (2.06–5.44)
Demographic and clinical variables, reperfusion/revascularization, adjunctive antiplatelet therapy	ref	1.24 (0.78–1.97)	1.56 (0.96–2.54)	3.45 (2.11–5.64)
+ **medication eligibility**				
Demographic and clinical variables, reperfusion/revascularization, adjunctive antiplatelet therapy, medication eligibility,	ref	1.24 (0.78–1.97)	1.56 (0.96–2.56)	3.56 (2.16–5.87)
+ **receipt of medications**				

GFR = glomerular filtration rate (ml/min/1.73 m2); ref = referent group

## Discussion

In this community-based population of patients with AMI, reduced GFR was common, associated with higher odds of 2-year mortality and yet lower use of guideline-based medications among eligible patients. However, in the two years following AMI, the excess mortality associated with severely reduced GFR persisted after accounting for variation in clinical and treatment factors. These results suggest that the underlying pathophysiology of kidney dysfunction is an important determinant of adverse outcomes in this population, and that improving guideline-based care in patients with kidney disease alone is not likely to close the substantial mortality gap in this patient population.

Because many clinical trials of AMI therapies exclude patients with significant kidney disease, the evidence base for treatment in this population derives primarily from observational studies, many of which have proposed that increasing treatment rates in patients with kidney disease will improve mortality.[6,9,11,12,17] Indeed, multiple studies have demonstrated under use of guideline-based therapies in patients with kidney disease.[1,2,6,9-13,18] While elevating the care for patients with kidney disease is an important goal, our results suggest that lower treatment rates alone do not account for the excess mortality. Our findings may differ from those of prior observational studies for several reasons. First, most studies evaluating the effectiveness of medical therapies did not account for eligibility for therapy,[6,12,17] which was explicitly collected in this prospective study. As the factors related to eligibility may be collinear with treatment, and ineligibility for treatment may be a marker for adverse outcomes, the failure to account for differences in eligibility is likely to result in residual confounding, which may over-estimate the effectiveness of treatments. Only a single study limited the evaluation of medical therapy to those who were ideal candidates for individual therapies in patients with and without end stage kidney disease.[11] However, this prior study did not adjust for reperfusion or revascularization, procedures that likely confound the relationship between mortality and medical treatment in AMI patients. PREMIER prospectively collected contraindications and intolerances to therapy and we adjusted for eligibility for each therapy. Second, intrinsic biases resulting from selection for angiography make it difficult to interpret the results of an effectiveness study in patients referred for coronary angiography.[9] PREMIER prospectively enrolled all patients with an AMI, minimizing such selection biases. Finally, many of the existing studies were performed over a decade ago, and thus do not reflect contemporary treatment for AMI. The current study was conducted in a contemporary cohort of AMI patients. Thus, more complete control of confounders, fewer selection biases and the use of contemporary data may account for the differences in the estimates of the impact of guideline-based therapies between this and prior studies.

The findings of this study suggest the need for a better understanding of the efficacy of established therapies for patients with AMI and kidney dysfunction. Despite the high and growing prevalence of kidney dysfunction in patients with AMI, randomized trials of therapies have generally excluded patients with more than mild kidney dysfunction, limiting available evidence for the efficacy of many therapeutic interventions in patients with more severe kidney dysfunction.[19] Current guideline recommendations for the use of aspirin and beta blockers are based on older trials which did not include representative numbers of patients with kidney dysfunction.[20,21] In fact, in a sample of published randomized controlled trials of therapies for AMI, 66% of trials excluded patients with kidney disease.[19] More recently, efforts have been made to evaluate the efficacy of statins and ACE inhibitors in patients with AMI and kidney dysfunction through sub-group analyses.[10,22] However, these studies are also limited by the exclusion criteria applied in the primary studies. It is crucial that future studies specifically target those with kidney dysfunction, or at the very least, include patients with all degrees of kidney dysfunction. Currently, for many therapies, observational studies provide the best evidence for treatment of AMI patients with kidney dysfunction.

Our finding that a broad range of clinical and treatment factors do not account for all of the increased risk of mortality associated with kidney dysfunction suggests that biologic mechanisms in kidney disease drive the higher mortality rates in this population. While these mechanisms likely contribute to all-cause mortality, the leading cause of death in patients with kidney dysfunction is cardiovascular disease.[7] Accordingly, further research to elucidate the biologic mechanisms of cardiovascular abnormalities in patients with kidney disease and develop novel therapies is needed. Several lines of evidence suggest mechanisms whereby kidney dysfunction may contribute a greater burden of cardiovascular disease. Lipid abnormalities, increased oxidative stress, inflammation, hyperhomocysteinemia and impaired nitric oxide bioavailability contribute to atherogenesis and may be more frequent or severe in kidney disease, resulting in accelerated atherogenesis.[7,8] Increased fibrinogen leads to a procoaguable state which may increase the likelihood of thrombotic events in patients with enhanced atherosclerosis.[8] In severe kidney dysfunction anemia, azotemia and abnormal calcium and phosphate metabolism may also play a role in cardiovascular pathogenesis.[7,23] These mechanisms suggest the potential for unique therapeutic approaches to improve outcomes in patients with kidney disease.

Certain factors should be considered in the interpretation of these results. We did not include follow-up medication data. Patients may have been started on guideline-based therapy after discharge from the hospital, or conversely, medications prescribed at the time of discharge may have been discontinued after discharge. This misclassification would tend to underestimate the relationship between therapies and outcomes. However, several studies suggest that the failure to implement therapy for patients with cardiovascular disease in the inpatient setting is a strong marker of the lack of outpatient therapy.[24] Also, we were not able to focus on the incremental benefit of additional therapies such as revascularization or other anti-platelet medications because contraindications to these therapies were not available. Therefore, we could not disentangle the issue of confounding by eligibility. However, we were able to assess a number of guideline-recommended therapies for which contraindications were documented. Furthermore, while some medication utilization rates were significantly lower in patients with kidney disease, treatment did not vary significantly for all treatments. Regardless, the substantial residual excess mortality in patients with severe kidney dysfunction suggests that current medical therapies are not sufficient to reduce mortality. In addition, contraindications were not precisely defined. However, contraindications as deemed present by the treating clinician were prospectively assessed. Because causes of death were not adjudicated, it was not possible to identify cardiovascular deaths, which are those likely to be reduced by guideline-based therapies. However, while chronic kidney disease is associated with an increased risk of non-cardiovascular mortality,[25] cardiovascular causes are leading causes of death in patients with kidney disease and AMI. Among patients with ESRD with AMI, cardiovascular causes account for more two thirds of deaths.[26] The estimated GFR during hospitalization may not reflect a steady state in some patients with AMI, and measures of albuminuria, an additional marker of chronic kidney disease, were not available. However, the risk for mortality was incrementally increased with worsening GFR, and persisted after adjustment for other clinical factors, consistent with the existing literature. Finally, as an observational study, unmeasured confounding may influence the results. However, we were able to adjust for a wide range of important measured demographic and clinical variables.

## Conclusion

In the two years following AMI, the excess mortality associated with kidney disease was not attributable to a wide range of clinical and treatment factors, including the under-use of guideline-based therapy. This study should not be interpreted as suggesting that guideline-based treatments in patients with AMI and kidney dysfunction are not effective. Certainly, there is a need for stronger evidence from randomized trials to inform the treatment of the large number of patients with kidney dysfunction and cardiovascular disease with existing treatments. However, these results suggest that the underlying physiology of kidney disease likely plays an important role in determination of outcomes. Therefore, novel therapies to treat patients with kidney dysfunction and cardiovascular disease are needed to substantially impact the high mortality observed for the large population of AMI patients with kidney dysfunction.

## Abbreviations

ACE Inhibitors: Angiotensin Converting Enzyme Inhibitor; ARBs: Angiotensin Receptor Blockers; CABG: Coronary Artery Bypass Graft; GFR: glomerular filtration rate; MI: myocardial infarction; NSTEMI: Non-ST-elevation myocardial infarction; PCI: Percutaneous Coronary Intervention; PREMIER: Prospective Registry Evaluating Myocardial Infarction: Event and Recovery; STEMI: ST-elevation myocardial infarction

## Competing interests

The authors declare that they have no competing interests.

## Authors' contributions

PP contributed to the conception and design of the study, interpretation of data, drafting and critical revision of the manuscript. AA contributed to the conception and design of the study and drafting and critical revision of the manuscript. PJ contributed to analysis of data, interpretation of data and drafting and critical revision of the manuscript. HK contributed to interpretation of data and drafting and critical revision of the manuscript. EK contributed to interpretation of data and drafting and critical revision of the manuscript. JS contributed to acquisition of data, interpretation of data and drafting and critical revision of the manuscript. JR contributed to the conception and design of the study and drafting and critical revision of the manuscript. FM contributed to the conception and design of the study, interpretation of data and drafting and critical revision of the manuscript. All authors read and approved the final manuscript.

## Pre-publication history

The pre-publication history for this paper can be accessed here:



## References

[B1] Masoudi FA, Plomondon ME, Magid DJ, Sales A, Rumsfeld JS (2004). Renal insufficiency and mortality from acute coronary syndromes. American Heart Journal.

[B2] Shlipak MG, Heidenreich PA, Noguchi H, Chertow GM, Browner WS, McClellan MB (2002). Association of renal insufficiency with treatment and outcomes after myocardial infarction in elderly patients. Ann Intern Med.

[B3] Al Suwaidi J, Reddan DN, Williams K, Pieper KS, Harrington RA, Califf RM (2002). Prognostic Implications of Abnormalities in Renal Function in Patients With Acute Coronary Syndromes. Circulation.

[B4] Smith GL, Shlipak MG, Havranek EP, Foody JM, Masoudi FA, Rathore SS (2006). Serum Urea Nitrogen, Creatinine, and Estimators of Renal Function: Mortality in Older Patients With Cardiovascular Disease. Arch Intern Med.

[B5] McClellan WM, Langston RD, Presley R (2004). Medicare Patients with Cardiovascular Disease Have a High Prevalence of Chronic Kidney Disease and a High Rate of Progression to End-Stage Renal Disease. J Am Soc Nephrol.

[B6] Wright RS, Reeder GS, Herzog CA, Albright RC, Williams BA, Dvorak DL (2002). Acute Myocardial Infarction and Renal Dysfunction: A High-Risk Combination. Ann Intern Med.

[B7] Dennis VW (2005). Coronary heart disease in patients with chronic kidney disease. J Am Soc Nephrol.

[B8] Best PJ, Reddan DN, Berger PB, Szczech LA, McCullough PA, Califf RM (2004). Cardiovascular disease and chronic kidney disease: insights and an update. American Heart Journal.

[B9] Ezekowitz J, McAlister FA, Humphries KH, Norris CM, Tonelli M, Ghali WA (2004). The association among renal insufficiency, pharmacotherapy, and outcomes in 6,427 patients with heart failure and coronary artery disease. Journal of the American College of Cardiology.

[B10] Anavekar NS, McMurray JJ, Velazquez EJ, Solomon SD, Kober L, Rouleau JL (2004). Relation between renal dysfunction and cardiovascular outcomes after myocardial infarction. New England Journal of Medicine.

[B11] Berger AK, Duval S, Krumholz HM (2003). Aspirin, beta-blocker, and angiotensin-converting enzyme inhibitor therapy in patients with end-stage renal disease and an acute myocardial infarction. Journal of the American College of Cardiology.

[B12] McCullough PA, Sandberg KR, Borzak S, Hudson MP, Garg M, Manley HJ (2002). Benefits of aspirin and beta-blockade after myocardial infarction in patients with chronic kidney disease. American Heart Journal.

[B13] Han JH, Chandra A, Mulgund J, Roe MT, Peterson ED, Szczech LA (2006). Chronic kidney disease in patients with non-ST-segment elevation acute coronary syndromes. American Journal of Medicine.

[B14] Spertus JA, Peterson E, Rumsfeld JS, Jones PG, Decker C, Krumholz H (2006). The Prospective Registry Evaluating Myocardial Infarction: Events and Recovery (PREMIER) – Evaluating the impact of myocardial infarction on patient outcomes. American Heart Journal.

[B15] Stevens LA, Coresh J, Greene T, Levey AS (2006). Assessing Kidney Function – Measured and Estimated Glomerular Filtration Rate. N Engl J Med.

[B16] (2002). K/DOQI clinical practice guidelines for chronic kidney disease: evaluation, classification, and stratification. Am J Kidney Dis.

[B17] Frances CD, Noguchi H, Massie BM, Browner WS, McClellan M (2000). Are we inhibited? Renal insufficiency should not preclude the use of ACE inhibitors for patients with myocardial infarction and depressed left ventricular function. Arch Intern Med.

[B18] Schiele F, Legalery P, Didier K, Meneveau N, Seronde MF, Caulfield F (2006). Impact of renal dysfunction on 1-year mortality after acute myocardial infarction. American Heart Journal.

[B19] Coca SG, Krumholz HM, Garg AX, Parikh CR (2006). Underrepresentation of Renal Disease in Randomized Controlled Trials of Cardiovascular Disease. JAMA.

[B20] (1988). Secondary prevention of vascular disease by prolonged antiplatelet treatment. Antiplatelet Trialists' Collaboration. British Medical Journal Clinical Research Ed.

[B21] (1985). Metoprolol in acute myocardial infarction (MIAMI). A randomised placebo-controlled international trial. The MIAMI Trial Research Group. Eur Heart J.

[B22] Tonelli M, Moye L, Sacks FM, Kiberd B, Curhan G, Cholesterol Recurrent Events (CARE) Trial Investigators (2003). Pravastatin for secondary prevention of cardiovascular events in persons with mild chronic renal insufficiency. Ann Intern Med.

[B23] Schrier RW (2006). Role of diminished renal function in cardiovascular mortality: marker or pathogenetic factor?. Journal of the American College of Cardiology.

[B24] Butler J, Arbogast PG, Daugherty J, Jain MK, Ray WA, Griffin MR (2004). Outpatient utilization of angiotensin-converting enzyme inhibitors among heart failure patients after hospital discharge. Journal of the American College of Cardiology.

[B25] Fried LF, Katz R, Sarnak MJ, Shlipak MG, Chaves PH, Jenny NS (2005). Kidney function as a predictor of noncardiovascular mortality. J Am Soc Nephrol.

[B26] Herzog CA, Ma JZ, Collins AJ (1998). Poor long-term survival after acute myocardial infarction among patients on long-term dialysis. N Engl J Med.

